# Modulation of Osteogenesis and Angiogenesis Activities Based on Ionic Release from Zn–Mg Alloys

**DOI:** 10.3390/ma15207117

**Published:** 2022-10-13

**Authors:** Ziming Wang, Weidan Wang, Xiuzhi Zhang, Fang Cao, Tianwei Zhang, Durga Bhakta Pokharel, Di Chen, Junlei Li, Jiahui Yang, Chi Xiao, Yuping Ren, Gaowu Qin, Dewei Zhao

**Affiliations:** 1 Department of Orthopaedics, Affiliated Zhongshan Hospital of Dalian University, Dalian 116001, China; 2 Institute of Metal Research, Chinese Academy of Sciences, Shenyang 110016, China; 3 Department of Biomedical Engineering, Dalian University of Technology, Dalian 116024, China; 4 School of Mechanical Engineering, Dalian Jiaotong University, Dalian 116028, China; 5 School of Materials Science and Engineering, Northeastern University, Shenyang 110819, China

**Keywords:** bone regeneration, microenvironment, Mg ion, synergistic effect, Zn ion

## Abstract

The enhancement of osteogenesis and angiogenesis remains a great challenge for the successful regeneration of engineered tissue. Biodegradable Mg and Zn alloys have received increasing interest as potential biodegradable metallic materials, partially due to the biological functions of Mg^2+^ and Zn^2+^ with regard to osteogenesis and angiogenesis, respectively. In the present study, novel biodegradable Zn–xMg (x = 0.2, 0.5, 1.0 wt.%) alloys were designed and fabricated, and the effects of adding different amounts of Mg to the Zn matrix were investigated. The osteogenesis and angiogenesis beneficial effects of Zn^2+^ and Mg^2+^ release during the biodegradation were characterized, demonstrating coordination with the bone regeneration process in a dose-dependent manner. The results show that increased Mg content leads to a higher amount of released Mg^2+^ while decreasing the Zn^2+^ concentration in the extract. The osteogenesis of pre-osteoblasts was promoted in Zn–0.5Mg and Zn–1Mg due to the higher concentration of Mg^2+^. Moreover, pure Zn extract presented the highest activity in angiogenesis, owing to the highest concentration of Zn^2+^ release (6.415 μg/mL); the proliferation of osteoblast cells was, however, inhibited under such a high Zn^2+^ concentration. Although the concentration of Zn ion was decreased in Zn–0.5Mg and Zn–1Mg compared with pure Zn, the angiogenesis was not influenced when the concentration of Mg in the extract was sufficiently increased. Hence, Mg^2+^ and Zn^2+^ in Zn–Mg alloys show a dual modulation effect. The Zn–0.5Mg alloy was indicated to be a promising implant candidate due to demonstrating the appropriate activity in regulating osteogenesis and angiogenesis. The present work evaluates the effect of the Mg content in Zn-based alloys on biological activities, and the results provide guidance regarding the Zn–Mg composition in designs for orthopedic application.

## 1. Introduction

Bone defects have become the most universal skeletal disorder in public health around the world. Bone is a highly vascularized tissue, with vessel networks providing access to delivered nutrients and oxygen, as well as secreting cytokines that regulate a series of biological activities [1,2,3]. Consequently, the bone remolding process requires the coupling of both osteogenesis and angiogenesis. The orthopedic implantation materials which are currently in clinical use include biological bone materials, bioceramics, synthetic polymers, and biomedical metal materials.

Of the various bone defect treatments, autologous bone transplantation, is considered the best choice since it can provide abundant blood supply while avoiding immune rejection by the body caused by allogeneic bone and xenogeneic bone. However, the supply of autologous bone grafts is limited and carries a risk of secondary injury and infection [1]. Alternative bone repair materials also have disadvantages. For example, the low elastic modulus of bioceramics and synthetic polymers limits their mechanical support for load-bearing sites in bone defect areas [4]. Moreover, the potential use of inert metallic materials in bone repair, despite having sufficient mechanical properties, is compromised by their lack of osteogenic and osteoinductive activities [5,6]. In addition, the elastic modulus of most biomedical metals is much higher than that of cortical bone, which can cause stress shielding or the loss of bone mass [2]. Meanwhile, the removal of these nondegradable metallic biomedical materials causes secondary damage and poses an economic burden to patients.

In recent years, biodegradable metals such as Mg, Fe, and Zn have been proposed and undergone development for clinical applications. In the past decade, iron (Fe) and magnesium (Mg) in both pure and alloyed states have been extensively studied as potential biodegradable metals [2,7]. Zinc-based alloys have been recognized as a new type of biodegradable metal, since Zn alloys exhibit moderate degradation rates faster than those of Fe and its alloys, but slower than those of Mg and its alloys [2]. However, the mechanical strength of pure Zn is insufficient for load-bearing applications [8,9]. As reported in recent studies, a good combination of strength and ductility can be achieved by the addition of 0.15 to 3 wt.% Mg [10,11,12]. Furthermore, regardless of Mg content, corrosion rates have been shown to be significantly lower for Zn–Mg alloys than for Mg alloys with corrosion evaluations [11,13]. However, the biological effects of different Mg contents in Zn matrices on bone regeneration have not been clarified, including details of the synergistic effect on osteogenesis and angiogenesis resulting from different amounts of Mg addition [14,15].

**Figure 1:**
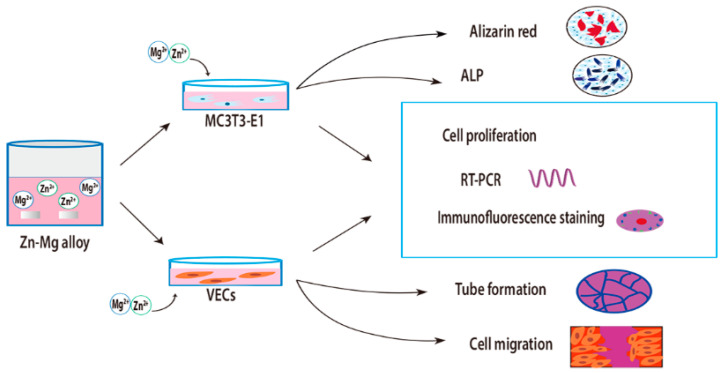
Schematic diagrams of the experimental design and protocol.

In this work, Zn–xMg (x = 0.2, 0.5, 1.0 wt.%) alloys were studied from the perspective of cellular biological activities, with pure Zn as the control group. Moreover, osteogenesis and angiogenesis properties were evaluated based on different contents of Mg addition to determine the appropriate composition of Zn–Mg alloys for use as biodegradable orthopedic implants. The expression levels of genes, which are important in the early and late stages of development of osteoblasts and endothelial cells, as well as the dose-dependent synergistic effect of Zn^2+^ and Mg^2+^, were also investigated for the first time. Figure 1 is a schematic diagram of experimental design and scheme.

## 2. Materials and Methods

### 2.1. Microstructure Characterization

In the current research, Zn–xMg (x = 0.2, 0.5, 1.0 wt.%) alloys were prepared by indirect extrusion, as previously reported [16]. The investigated pure Zn and Zn–Mg alloys were cut using a diamond-blade saw to obtain longitudinal sections. After grinding and polishing, all samples were etched in a mixture solution of hydrochloric acid and distilled water. The evolution of the extruded microstructure was assessed by scanning electron microscopy (SEM, Hitachi 3500, Tokyo, Japan).

### 2.2. Preparation of Extract Solution

An in vitro degradation process can be performed in a variety of solutions that mimic human body fluids [5,17,18,19]. In order to evaluate the effects of different proportions of Zn–Mg alloys in osteogenic cells and endothelial cells, different proportions of Zn–Mg alloy extract solutions were prepared in minimum essential medium alpha modification (α-MEM, Hyclone, Logan, UT, USA) for subsequent testing. Samples of zinc–magnesium alloys of the same shape (10 mm in diameter and 3 mm in height) were sterilized by immersion in 75% ethanol and the samples were irradiated for 30 min from both the front and back sides using ultraviolet rays. Extracts of the sample alloys were prepared according to ISO 10993-5 standards [20] with an extraction ratio of 1.25 cm^2^/mL. The extract applied for all tests was collected following 24 h of extraction. In order to reduce errors in the ionic concentration between different batches of extracts, the extract for each alloy was extracted multiple times under the same conditions. The complete medium was in a ready-to-use form before each experiment.

### 2.3. Ion Concentration Detection

The Mg ion concentration of each extract was characterized with ICP-OES (iCAP 6500, Thermo Fisher Scientific, Waltham, MA, USA), while α-MEM without sample immersion was set as the control group, which was subjected to the same incubation conditions as the experimental group.

### 2.4. Cell Culture

Murine pre-osteoblast cells (MC3T3-E1, ATCC) were cultured in 10% fetal bovine serum (FBS, Gibco, NY, USA), 100 U/mL penicillin, and 100 μg/mL streptomycin in α-MEM in a cell culture incubator containing 5% CO_2_ at 37 °C. Mouse vascular endothelial cells (VECs, ATCC) were cultured in ECM medium (ScienCell Research, Carlsbad, CA, USA) supplemented with growth factors, 5% fetal bovine serum, and 1% penicillin–streptomycin, and incubated at 37 °C with 5% CO_2_. Cells were passaged every 3 days, and the medium was renewed every 2 days, and cells from generations 5 to 7 were used for experimental studies. The Figure 1 is the schematic diagram of the experimental design and protocol.

### 2.5. Cell Proliferation Experiments

A cell counting kit-8 (CCK-8, Dojindo, Kumamoto, Japan) was used for measuring cell proliferation. MC3T3-E1 (5 × 10^3^) and VEC (3 × 10^3^) cells were added to wells of 96-well plates, with 5 parallel wells set per test from which the average data were obtained. The plates were cultured at 37 °C in a CO_2_ incubator. After the cells adhered to the well, the liquid was exchanged, and 100 μL of Zn–Mg alloy α-MEM extract was added per well for culturing for 1, 3, and 5 days. Then, 10 μL per well of CCK-8 working solution was added to the medium followed by incubation for an additional 2 h at 37 °C. Absorbance at 450 nm was then recorded using a microplate reader (Bio-Tek, Winooski, VT, USA).

### 2.6. In Vitro Osteogenesis Differentiation Test

Staining with alizarin red and alkaline phosphatase (ALP) was performed for the abovementioned extract. For the alizarin red assay, 8 × 10^4^ cells were inoculated in each well of a 6-well plate, with each group of dip extraction containing 10 mM β-glycerophosphate, 100 nM dexamethasone, and 50 mM ascorbate culture with incubation for 14 and 21 days. Subsequently, cells were fixed with 4% paraformaldehyde for 30 min at room temperature, followed by staining with alizarin red solution for 1 h. The red stained area was observed using an optical microscope (Olympus, Tokyo, Japan). For the ALP assay, MC3T3-E1 cells of the same density were inoculated in each well of a 6-well plate, and the dip extraction of each group contained 10 mM β-glycerophosphate, 100 nM dexamethasone, and 50 mM ascorbate culture with incubation for 7 and 14 days. The BCIP/NBT Alkaline Phosphatase Color Development Kit (Beyotime, Haimen, China) was used in the subsequent tests, and the purple-stained area was recognized as a positive mineralization result.

### 2.7. In Vitro Angiogenic Differentiation Test

Tube formation and cellular wound-healing assays were performed on VECs with extract co-culture to evaluate the effects of different proportions of zinc–magnesium alloys on angiogenic differentiation. For the tube formation assay, Matrigel (Corning, NY, USA) was thawed overnight at 4 °C, and the tip pipette and 96-well culture plate were pre-cooled at −20 °C for 24 h. Thereafter, 50 μL of thawed Matrigel was spread on each well of a cooled 96-well plate. Five parallel samples were set up for each group, and the plate was incubated at 37 °C for 2 h for conversion to gel form. Subsequently, 100 μL extraction was mixed with VECs for every well with 6 × 10^3^ cells, and added to each well and incubated for 3 h. The tube formation was observed under a light microscope, followed by imaging at one-hour intervals. Image-Pro Plus software was applied for quantitative analysis. Except for during observation time, the cells remained in a cell culture incubator containing 5% CO_2_ at 37 °C.

For wound-healing tests, 2.5 × 10^4^ VEC cells were cultured in each well for migration assays (Ibidi, Madison, WI, USA) for 12 h with 80 μL of liquid per well, and the inserts in the well plate were removed when the cells were adherent, and the medium was then replaced with 800 μL of the extract solution. The initial distance of the scratch was imaged at 0 h after the liquid exchange, and the migrated cells were imaged with a light microscope after 0 and 12 h. The cells were placed in a cell culture incubator containing 5% CO_2_ at 37 °C, except for during observation and imaging.

### 2.8. Real-Time Quantitative Polymerase Chain Reaction (RT-qPCR) Analysis

MC3T3-E1 was inoculated using 4 × 10^4^ in each well and incubated with the extract solution in 6-well plates for 14 and 21 days. The same amount of VECs in each well was incubated with extract solution on 6-well plates for 3 and 7 days. Osteogenesis-related genes (ALP, RUNX2, OCN, and COL-1) and angiogenesis-related genes (VEGF, HIF-1α, CD31, and vWF) were analyzed with RT-qPCR. MC3T3-E1 at 14 and 21 days and VECs at 3 and 7 days were processed using TRIzol reagent (Invitrogen, Waltham, MA, USA). RNA was extracted from cells using chloroform, precipitated with isopropanol, and finally washed with 75% ethanol. The RNA was used for reverse transcription into complementary DNA (cDNA) using a hypermix kit (TaKaRa, Beijing, China). The obtained cDNA was mixed with SYBR Green Mastermix and primers and subject to the PCR loop program. Gene expression was analyzed with the 2^−ΔΔCt^ method.

### 2.9. Immunofluorescence Staining

MC3T3-E1 cells were fixed for 15 min with paraformaldehyde and lysed with 0.3% Triton X for 20 min. Then, 1% bovine serum albumin (BSA, Solarbio, Beijing, China) was used to block for 20 min. Cells were treated overnight at 4 °C with the primary antibody (Abcam, anti-RUNX2, Cambridge, UK). The next day, samples were washed and incubated with the secondary antibody (Abcam, Alexa Fluor 594, Cambridge, UK) for 75 min at room temperature. After rinsing with PBS, they were stained with phalloidin (Yeasen, Shanghai, China) and DAPI (Byotime, Haimen, China) for 20 min. Samples were then imaged using confocal microscopy (LSM 9, ZEISS, Dresden, Germany). As for VSCs, cells were treated according to the same process as for MC3T3-E1 with paraformaldehyde fixation for 15 min and lysis with 0.3% Triton X for 20 min. After blocking for 20 min, cells were incubated with the primary antibody (Abcam, anti-VEGF, UK) overnight at 4 °C. After rinsing the cells the next day, the secondary antibody (Abcam, IgG-Alexa Fluor 594, UK) was incubated at room temperature for 75 min, followed by protection from light to avoid fluorescence quenching. After rinsing with PBS, VECs were stained with phalloidin and DAPI for 20 min, as described above. Fluorescence images were captured by confocal microscopy, and the fluorescence area was quantitatively analyzed.

### 2.10. Statistical Analysis

The data are reported as mean ± SD for three replicates. An unpaired *t*-test was used to conduct comparisons between the two groups. GraphPad Prism version 9 was used, followed by a one-way ANOVA for Tukey multiple comparison. Significance levels were set to *p*-value < 0.05. An asterisk (*) indicates a significant difference at *p* < 0.05; (**) indicates a significant difference at *p* < 0.01; (***) indicates a significant difference at *p* < 0.001; and (****) indicates a significant difference at *p* < 0.0001.(ns) indicates no statistical significance.

## 3. Results

### 3.1. Microstructure

**Figure 2:**
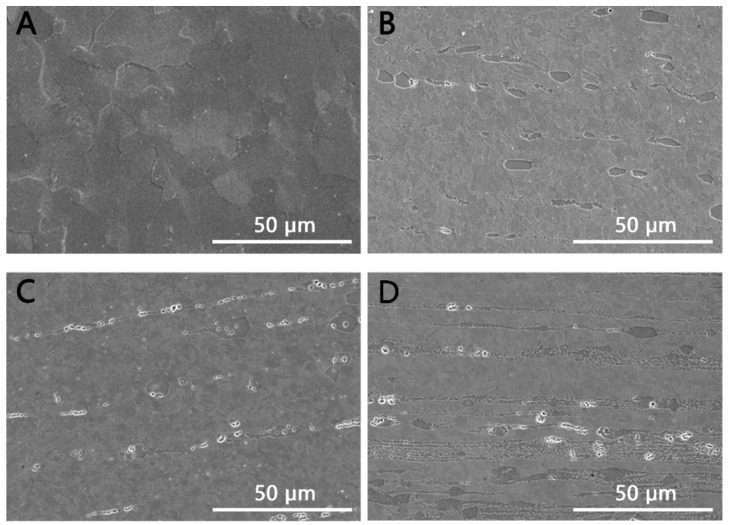
SEM morphologies of the longitudinal section of the as-extruded Zn–Mg alloys. (**A**) Pure Zn; (**B**) Zn–0.2Mg; (**C**) Zn–0.5Mg; (**D**) Zn–1Mg.

As observed in Figure 2C,D the lengthened morphology in the Zn–0.5Mg and Zn–1Mg alloys is due to broken up intermetallic compounds distributed along the extrusion direction, which was caused by the hot extrusion process. The main secondary phase in Zn–xMg (x = 0.2, 0.5, 1.0 wt.%) alloys was the Mg_2_Zn_11_ phase, as shown in previous work [10,16,21]. This clearly shows that the volume fraction of the Mg_2_Zn_11_ phase increases with an increase in Mg content. Moreover, secondary-phase particles play an important role in the refinement of grain size.

### 3.2. Ion Concentration Measurement

**Figure 3:**
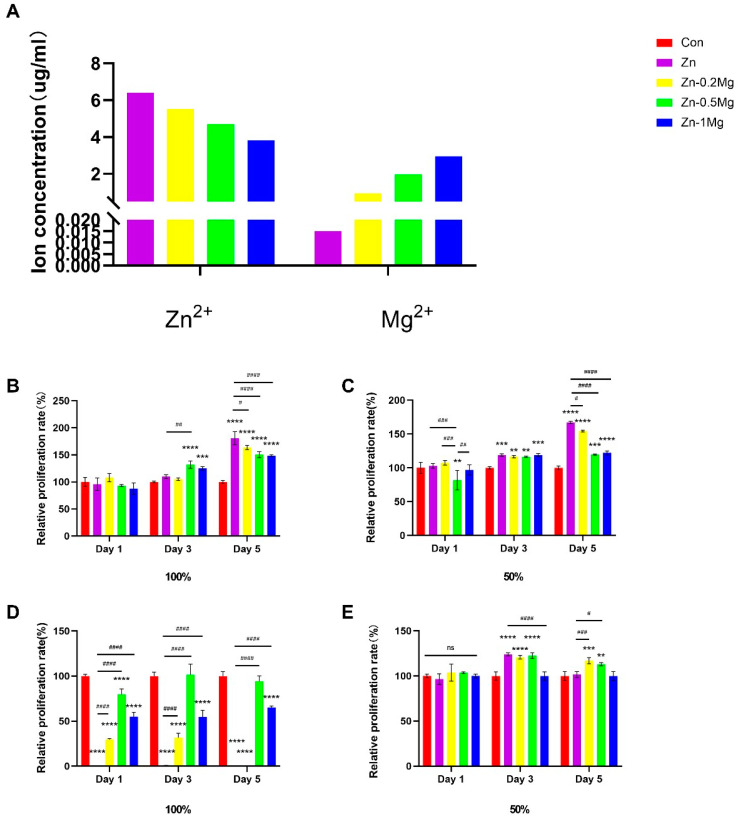
(**A**) Results of Zn^2+^ and Mg^2+^ release, measured with ICP-OES in α-MEM, and relative proliferation rate of (**B**,**C**) VECs incubated in original and two-fold dilution extract; (**D**,**E**) MC3T3-E1 cells incubated in original and two-fold dilution extract. Compared with the control group, **** *p* < 0.0001, *** *p* < 0.001 and ** *p* < 0.01; compared with the Zn group, #### *p* < 0.0001, ### *p* < 0.001, ## *p* < 0.01, and # *p* < 0.05; (ns) indicates no statistical significance.

The concentration of Zn^2+^ and Mg^2+^ released from pure Zn and Zn–Mg alloys was determined according to ISO 10993-5 and measured with ICP-OES. The Zn^2+^ and Mg^2+^ concentrations presented in Figure 3A were calculated after the deduction of the original ion concentrations in the medium of Zn^2+^ and Mg^2+^, which were 0.015 ± 0.00125 and 19.86 ± 0.1015 μg/mL, respectively. It can be seen from Figure 3A that with the increase in Mg content of the alloy material, there is a decrease in the concentration of Zn^2+^ and an increase in the concentration of Mg^2+^ released in the medium.

### 3.3. Cell Proliferation Experiments

It can be seen from the cell proliferation results in Figure 3B,C that there was no statistically significant differences between the VECs incubated with each group at day 1 in the undiluted extract, while the Zn–0.5Mg and Zn–1Mg alloy groups showed higher proliferation compared with the control group at day 3 in contrast to the pure Zn group, which was not significantly different. At day 5, there was a significant enhancement in the proliferation rate of all Zn–Mg alloy groups compared with the blank control group. Among them, the highest proliferation was observed in the pure Zn group, followed by the Zn–0.2Mg alloy. As for the two-fold diluted extract, the proliferation of the Zn–0.5Mg group at day 1 was slightly lower than for the control group, while no statistically significant differences appeared among the other groups. Compared with the control group, pure Zn and Zn–Mg alloy groups revealed an increased proliferation rate at day 3 (*p* < 0.05). Concurrently, day 5 showed a similar variation in proliferation to day 3, and pure Zn extract showed significant differences from each other group. A slight decrease was observed when incubated with the diluted extracts.

As shown in Figure 3D,E, after the co-culture of MC3T3-E1 with pure Zn and Zn–Mg alloy extracts, the proliferation of MC3T3-E1 cells was inhibited in the pure Zn group in the undiluted extract and significantly decreased in the Zn–Mg alloy group compared with the control group (*p* <0.05), while there was an increase in the proliferation rate for cells of the Zn–0.5Mg alloy group on the third day, indicating their similarity to the control group. The proliferation trends for Zn–Mg groups at day 5 were similar to those of day 1 and 3. Moreover, after the extracted solution was diluted to 50% concentration, similarly to that of the original extract with α-MEM, no statistically significant differences were observed between any of the groups at day 1. Even so, the Zn–Mg alloy group demonstrated enhanced proliferation compared with the control group at day 3, while no obvious difference was apparent when comparing pure Zn with the control group. At day 5, Zn–0.5Mg showed significantly higher proliferation compared with the control group (*p* < 0.05). Considering the inhibition effect of the 100% extract, subsequent experiments were performed with the two-fold (50%) diluted extract.

### 3.4. In Vitro Evaluation of Osteogenesis Differentiation

#### 3.4.1. Alizarin Red and ALP Activity Detection

**Figure 4:**
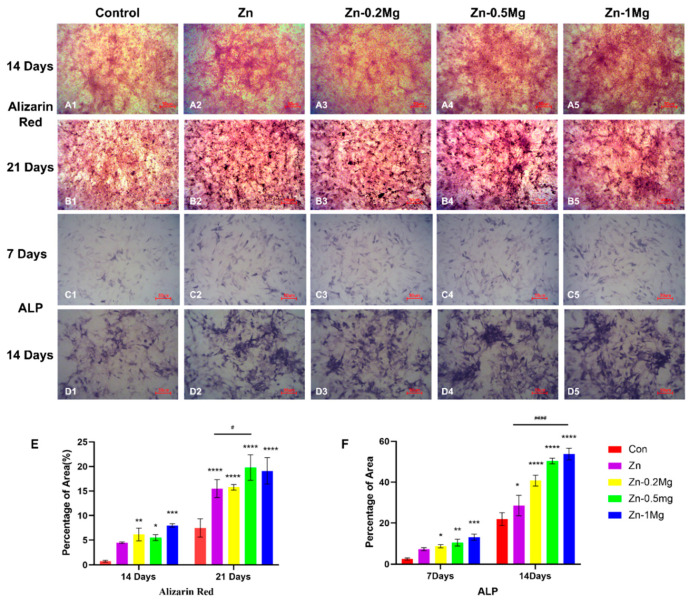
In vitro osteogenesis assessment (**A1**–**A**5 were pictures of alizarin red for 14 days, **B1**–**B**5 were pictures of alizarin red for 21 days, and **C1**–**C**5 were pictures of ALP for 7 days). Plots of MC3T3-E1 cultured with various extracts for (**A**,**B**) alizarin red at 14 and 21 days, and (**C**,**D**) alkaline phosphatase (ALP) at 7 and 14 days (**E**,**F**) along with corresponding quantitative results. Compared with the control group, **** *p* < 0.0001, *** *p* < 0.001, ** *p* < 0.01, and * *p* < 0.05; compared with the Zn group, #### *p* < 0.0001, and # *p* < 0.05.

Figure 4A,B show the results of alizarin red staining at 14 and 21 days. At 14 days, there was no significant difference between the experimental groups, and it was notable that, compared with the control group (0.738 ± 0.0.191), the difference and its significance were the highest (*p* < 0.0001) for Zn–1Mg (7.977 ± 0.339) and relatively lower for pure Zn (4.474 ± 0.614) (*p* < 0.05). The red stained area of the cells in the Zn–0.5Mg (19.777 ± 2.599) and Zn–1Mg groups (19.102 ± 2.698) were significantly enhanced at 21 days compared with the control group (7.473 ± 1.861). Meanwhile, the pure Zn (15.510 ± 1.833) and Zn–0.2Mg groups (15.783 ± 0.568) had obvious calcium deposition and the formation of calcium nodules was visible compared with the images at 14 days. The ALP staining results presented in Figure 4C,D show a significant difference between the Zn–Mg alloy groups and the control group at 7 days (*p* < 0.05), while pure Zn (7.272 ± 0.695) was not significantly different from the control group (2.56 ± 0.524). As for the results of 14 days, there was a statistically significant difference between pure Zn (28.587 ± 4.993) and the control group (21.927 ± 3.151) (*p* < 0.05) and, moreover, the Zn–Mg alloy groups were also found to be significantly different from the control group (*p* < 0.0001).

#### 3.4.2. Effect of Materials on Osteogenesis Gene Expression

**Figure 5:**
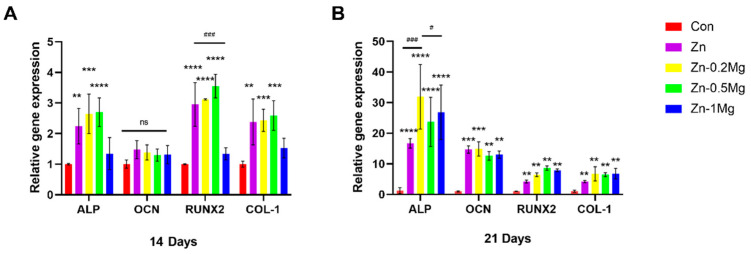
Expression of genes related to osteogenesis. Gene expression results for MC3T3-E1 at (**A**) 14 days and (**B**) 21 days. Compared with the control group, **** *p* < 0.0001, *** *p* < 0.001 and ** *p* < 0.01; compared with the Zn group, ### *p* < 0.001 and # *p* < 0.05; (ns) indicates no statistical significance.

Regarding the gene level, several osteogenesis markers were selected for analysis, including ALP, OCN, RUNX2, and Collagen I (COL-1), as shown in Figure 5. The results of Figure 5A show that the expression of ALP, RUNX2, and Col-I at 14 days were significantly higher in the Zn, Zn–0.2Mg, and Zn–0.5Mg groups than in the control group (*p* < 0.01). At day 21, all osteogenesis markers were significantly more highly expressed in the Zn and Zn–Mg alloy groups than in the control group (*p* < 0.01). All in all, the Zn–0.5Mg group demonstrated a significantly enhanced expression of osteogenesis markers at both 14 and 21 days.

#### 3.4.3. Immunofluorescence Analysis of Proteins Related to Osteogenesis

**Figure 6:**
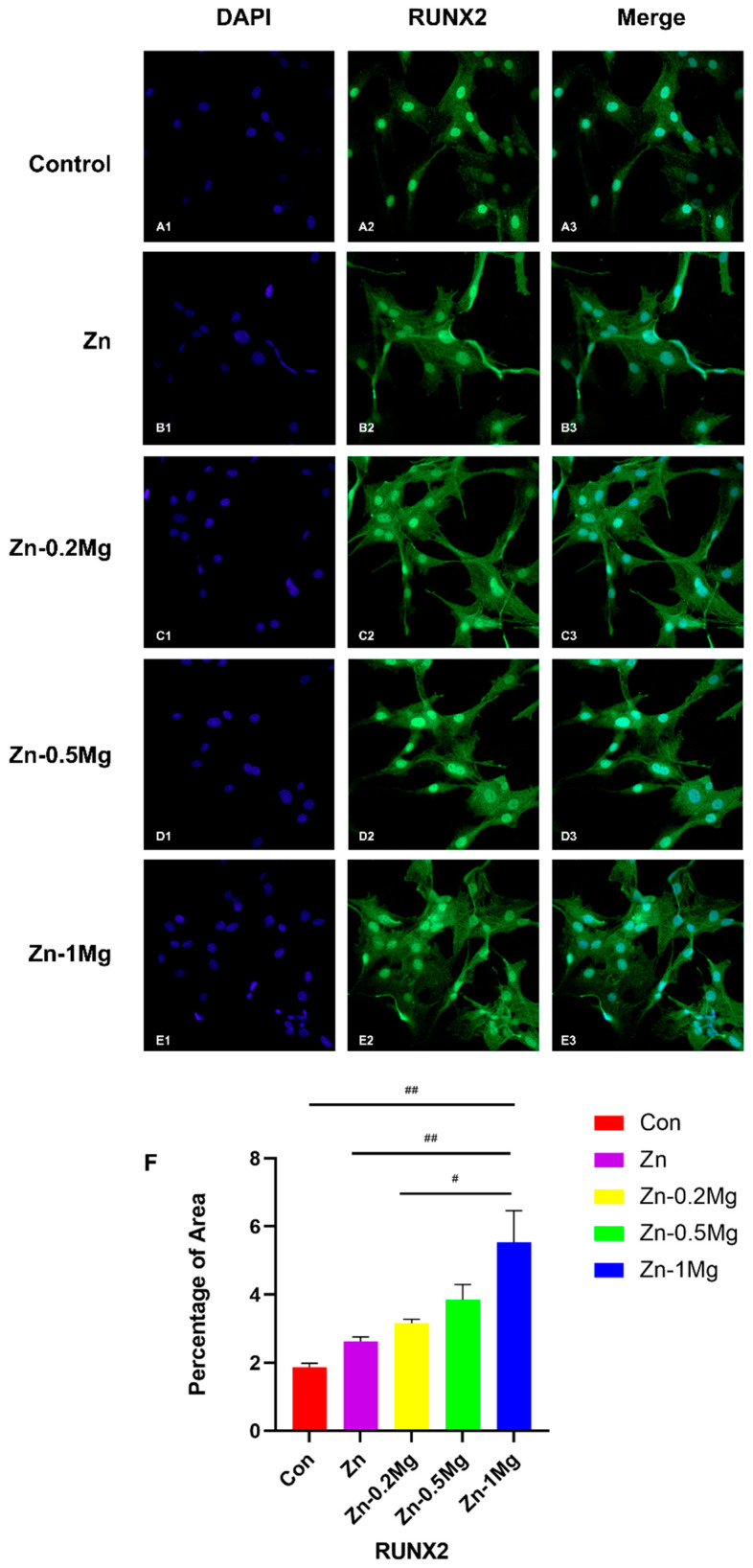
Representative immunofluorescence staining images of (**A1**–**A3** were the control group, **B1**–**B3** were the Zn group, **C1**–**C3** were the Zn-0.2Mg group, **D1**–**D3** were the Zn-0.5Mg group, **E1**–**E3** were the Zn-1Mg group) MC3T3-E1 and (**F**) the quantitative results. ## *p* < 0.01 and # *p* < 0.05, compared with Zn–1Mg group.

RUNX2 plays an important role in regulating bone-related genes, and regulates bone resorption, formation, and reconstruction processes via the regulation of osteoblasts, chondrocytes, osteoclasts, and other bone cells [22]. RUNX2 can also maintain the formation and differentiation of osteoblasts through related signaling pathways, and is one of the landmark genes of osteoblasts in the mineralization phase [23]. To evaluate the influence of materials on osteogenesis differentiation, osteoblasts of the same density were co-cultured with sample extract for 48 h and imaged using confocal microscopy. It can be seen in Figure 6 that more cells and relatively strong RUNX2 immunofluorescence labeling are observed for the cells cultured in the extract of Zn–Mg alloys, while the cell skeleton shows favorable cell-spreading morphology. According to the quantitative results in Figure 6F, RUNX2 protein expression was significantly higher in the Zn–1Mg alloy (5.535 ± 1.61%) compared with the other groups (*p* < 0.05), except Zn–0.5Mg (3.853 ± 0.7737%). Only a few cells were observed in the pure Zn (2.628 ± 0.2272%) and control groups (1.873 ± 0.196%), of which the immunofluorescence staining intensity of the cell skeleton was weak.

### 3.5. Evaluation of Angiogenesis Behavior In Vitro

In the process of bone trauma as well as bone transplantation, not only are characteristics of osteogenesis required but an abundant blood supply is also essential for providing sufficient nutrients to promote the generation and proliferation of bone cells. As a result, vascular formation in the early stages of bone trauma and bone grafting is crucial to the success of bone regeneration. Zn^2+^ and Mg^2+^ are reported to induce pro-angiogenesis in vitro [24,25]. Therefore, a series of experiments were conducted in the present work to evaluate the effects of Zn–Mg alloys on angiogenesis with the addition of different amounts of Mg.

#### 3.5.1. Tube-Formation Ability with Material Induction

**Figure 7:**
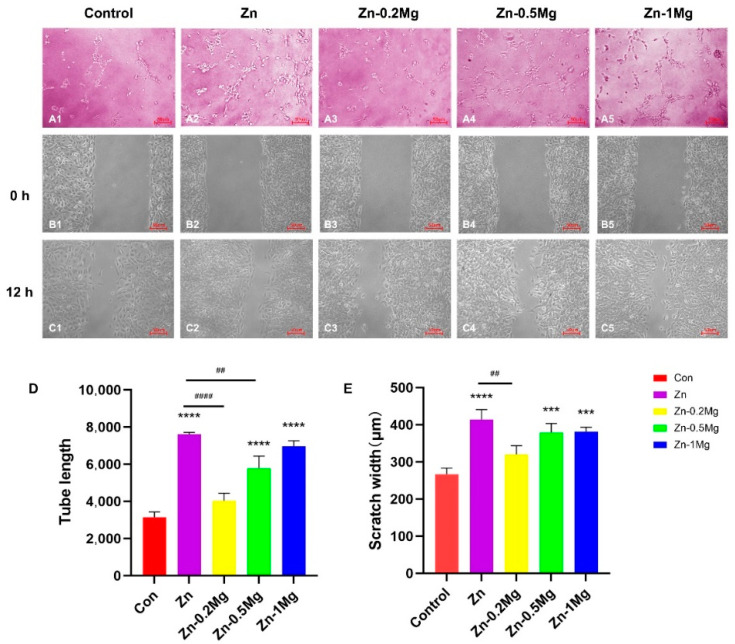
In vitro angiogenesis assessment. (**A1**–**A**5 were pictures of VECs forming vascular rings, **B1**–**B**5 were pictures of cell migration of MC3T3-E1 at 0h, **C1**–**C**5 were pictures of MC3T3-E1 at 12h) Quantification of tube formation tests and (**D**) corresponding tube lengths of various extract groups. The results of (**B**,**C**) the VEC migration test and (**E**) their migration width (um) after 12 h of incubation (**** *p* < 0.0001, *** *p* < 0.001, compared with the control group; #### *p* < 0.0001, ## *p* < 0.01 compared with the Zn group).

To understand the effects of Mg^2+^ and Zn^2+^ release from Zn–Mg alloys on the functioning of angiogenesis, a microtubule formation experiment was conducted using Matrigel and the total length of tube formation was quantitatively analyzed with ImageJ software version 1.8.0_172 was used (NHI, NY, USA) and through graphing. Figure 7A,D show the images and results of the VEC tube formation. At the beginning, the same total amount of VECs were uniformly seeded per well. After the VECs were cultured with either pure Zn or Zn–Mg alloy extract for 3 h, the VECs became connected, and tubular structures gradually appeared, which was particularly obvious for pure Zn (7616 ± 95.04 μm), Zn–0.5Mg (5776 ± 661.6 μm), and Zn–1Mg (6975 ± 288.9 μm) alloy extracts. Compared with the control group (3146 ± 162.2 μm) of the general complete medium, pure Zn, Zn–0.5Mg, and Zn–1Mg were significantly different (*p* < 0.0001), while more tube formation for Zn–0.2Mg (4038 ± 403.1 μm) was also recorded, though was not statistically significant. In addition, there were no significant differences between pure Zn and Zn–1Mg.

#### 3.5.2. Migration Ability with Material Induction

The migration of VECs is critical for neonatal angiogenesis. As shown in Figure 7B,C a wound scratch experiment was performed to investigate the ability of the extract to induce VEC migration, and a change in wound closure was recorded at 12 h. ImageJ was used to quantitatively analyze the scratch width. It can be seen from the wound healing experiment results that pure Zn (414.4 ± 26.81 μm), Zn–0.5Mg (378.8 ± 24.8 μm) and Zn–1Mg (381.4 ± 12.3 μm) alloy extracts had a wider migration width compared with the control group (266.9 ± 16.79) (*p* < 0.001); of these, pure Zn extract demonstrated the greatest effect in enhancing the migration of VECs (Figure 7E).

#### 3.5.3. Effect of Materials on Vascular Gene Expression

**Figure 8:**
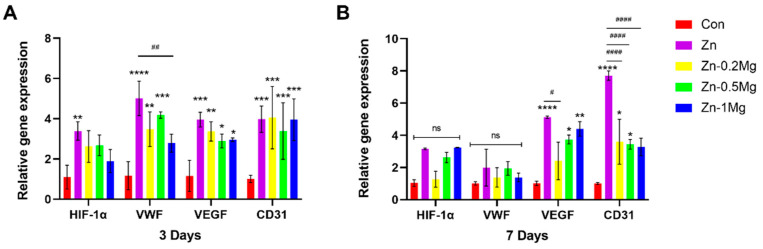
Expression of genes related to angiogenesis. Gene expression in VECs after (**A**) 3 days and (**B**) 7 days in various sample extracts (**** *p* < 0.0001, *** *p* < 0.001, ** *p* < 0.01, * *p* < 0.05 compared to the control group; #### *p* < 0.0001, ## *p* < 0.01, # *p* < 0.05 compared with the Zn group; (ns) indicates no statistical significance.).

Figure 8A,B show the expression of angiogenesis-related genes (HIF-1α, VWF, VEGF and CD31) after VECs were cultured for 3 and 7 days. Compared with the control group, the expression of vascular marker genes in each group had increased at 3 days, and the relative expression of the early vascular gene HIF-1α was higher in the pure Zn group than in the Zn–Mg alloy and control groups. Moreover, there were no significant differences in the expression of VWF, VEGF, and CD31 between pure Zn and the Zn–0.2Mg and Zn–1Mg alloys. However, the expression of angiogenesis genes was significantly enhanced in the pure Zn and Zn–Mg alloy groups compared with the control group (*p* < 0.05), except for HIF-1α in the Zn–Mg alloy extracts. At 7 days, the angiogenesis-related genes VEGF and CD31 were significantly expressed compared with the control group (*p* < 0.05), and the pure Zn group indicated the highest gene expression, while the expression of HIF-1α and vWF genes did not show significant differences between the groups.

#### 3.5.4. Immunofluorescence Analysis of Proteins Related to Angiogenesis

**Figure 9:**
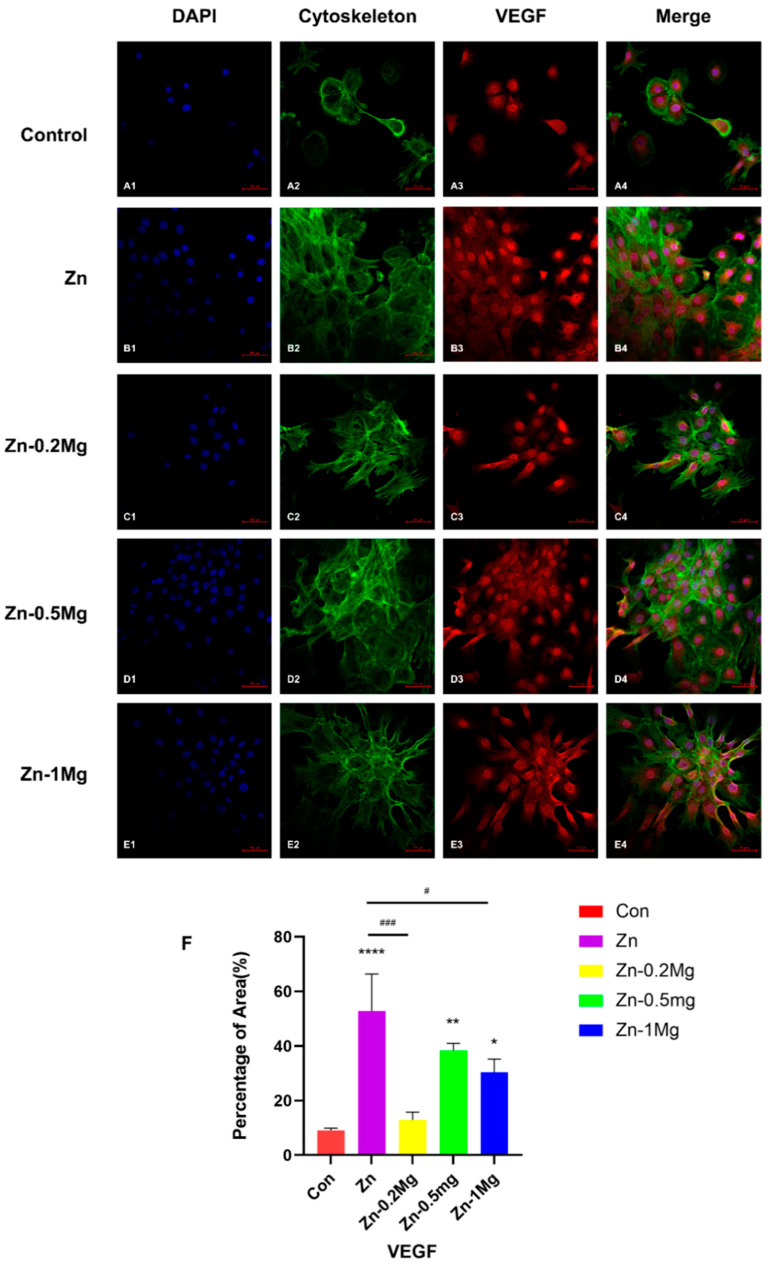
(**A1**–**A4** were the control group, **B1**–**B4** were the Zn group, **C1**–**C4** were the Zn-0.2Mg group, **D1**–**D4** were the Zn-0.5Mg group, **E1**–**E4** were the Zn-1Mg group) Representative immunofluorescence staining images and (**F**) quantitative analysis of VECs. Compared with the control group, **** *p* < 0.0001, ** *p* < 0.01, * *p* < 0.05; compared with Zn group, ### *p* < 0.001, # *p* < 0.05.

In order to evaluate the angiogenesis effect of each group of extracts on VECs, VECs of the same density were co-cultured with extracts for 48 h. VEC morphologies were recorded using confocal microscopy, and the immunofluorescence area under the same view was quantitatively analyzed using ImageJ software. Figure 9B shows that the pure Zn group (52.81 ± 13.54) has a large number of cells adhered on the confocal dish and the cytoskeleton can also be clearly observed, with similar but less pronounced changes in the Zn–0.5Mg (38.40 ± 2.545) and Zn–1Mg (30.46 ± 4.797) groups, while the immunofluorescence-stained area of the control group (9.073 ± 0.7991) was relatively low. It could be seen from Figure 9F that, except for Zn–0.2Mg (12.92 ± 2.842), VEGF protein expression was significantly extended (*p* < 0.05) in the extract groups compared with the control group, and that the highest value was observed in the pure Zn group (*p* < 0.0001).

## 4. Discussion

In areas of fracture injury with poor blood supply, fracture nonunion or delayed fracture often occurs due to ischemia at the fracture end, which seriously affects patient recovery. Studies have shown that osteogenesis and angiogenesis in the mammalian skeletal system are closely related to bone growth and regeneration in bone remodeling and help in maintaining bone homeostasis [26]. During angiogenesis, various growth factors involved in the coupling of angiogenesis and osteogenesis are released, such as platelet-derived growth factor BB (PDGF-BB), hypoxia-inducible factor 1-α (HIF-1α), Notch 1, and vascular endothelial growth factor (VEGF). Therefore, the performances of both angiogenesis and osteogenesis are frequently analyzed as a method for evaluating bone implants in orthopedic biomaterial research, namely, serving as indicators of superior biocompatibility and promoting bone formation.

In this study, the cell viability and performance of MC3T3-E1 and VECs co-cultured with extracts of pure Zn and Zn–xMg (x = 0.2, 0.5, 1.0 wt.%) alloys were investigated. The results show that the two-fold diluted extract of pure Zn and Zn–Mg alloys could enhance the proliferation and expression of related genes in MC3T3-E1. Likewise, the cell viability of VECs and the angiogenesis performance were also enhanced in the two-fold diluted extract. However, for MC3T3-E1 cells co-cultured with original pure Zn and Zn–0.2Mg extracts, the proliferation of osteoblasts was significantly inhibited. Similar results have been reported in Yang et al. [27,28], where high concentrations of Zn^2+^ demonstrated toxic effects on osteoblasts. Studies have reported that low concentrations of Zn^2+^ may promote the activity of osteoblasts, endothelial cells, and vascular smooth muscle cells, while high concentrations of Zn^2+^ decrease the activity of these cells [29,30,31]. This may be related to the role of Zn^2+^ in regulating actin polymerization and depolymerization, because actin is required in cell proliferation and differentiation for cytoskeleton polymerization toward maintaining the stability of morphological structure, while cell apoptosis will result in cell membrane function loss, cell edema, cell fragmentation, and the formation of apoptotic bodies [32,33,34,35]. Ma et al. found that Zn^2+^ promotes the expression of stress fibers at 60 μM but inhibits their expression at 140 μM [30]. Therefore, Zn^2+^ has a dose-dependent effect on cell viability, proliferation, adhesion, and migration.

The method of extract dilution (50% concentration of the original extract) was used in this study to test osteoblasts and endothelial cells. Considering that simulation is difficult even for materials that have the same composition and preparation process, the simulation of complex in vivo physiological conditions under an in vitro environment is especially challenging. Thus, different degradation rates are reported between in vitro and in vivo studies. The local concentration of zinc and magnesium ions in the body can be reduced by the circulation of body fluids, the difference of which can refer to the relevant research on degradable magnesium-based implants. Wang et al. reviewed the differences in magnesium ions between the sensitivity of cells in degradable magnesium-based implants in vitro and in vivo [36], and suggested that the original extract should be diluted by 6–10 times to evaluate the in vitro cytotoxicity of degradable magnesium-based implants, so as to help to determine the potential health risks of degradable magnesium-based biomaterials. Moreover, Sanchez et al. discussed the systematic correlation between the in vitro and in vivo degradation of magnesium alloys [37], concluding the in vivo corrosion rate was possibly 1–4 times lower than that found in vitro. The range could be further reduced to 1–3 times if the selected medium can sufficiently simulate the physiological conditions. However, no specific studies have been carried out examining the difference in in vivo and in vitro corrosion rates for biodegradable zinc alloys. In short, a two-fold diluted extract was applied to all experimental groups, and the tendency of Zn^2+^ and Mg^2+^ release during osteogenesis and angiogenesis was evaluated.

According to this study, the extract of Zn–0.5Mg exhibited a relatively good proliferative effect on MC3T3-E1 in both undiluted and two-fold-diluted extract medium, which could be related to the interaction between Zn^2+^ and Mg^2+^ according to their specific ratios. In terms of VECs, it could be seen that in both undiluted and two-fold-diluted extract medium, the proliferation rates were higher for pure Zn and Zn–0.2Mg extract than the other three groups due to the fact that endothelial cells are more tolerant of zinc ions than osteoblasts, which is consistent with the findings reported by Yang et al. [28].

In osteogenesis, the activities of alizarin red and alkaline phosphatase (ALP) in the Zn–0.5Mg and Zn–1Mg alloy groups were significantly enhanced (Figure 4A,B), and the expression of osteogenesis-related genes (ALP, Col I, OCN, and RUNX-2) was significantly upregulated (Figure 5A,B). After the two-fold extract co-culturing, alizarin red calcium nodules and calcium deposition were stronger in the pure Zn group than in the blank control group. In addition, the alkaline phosphatase activity and the expression levels of osteogenesis-related genes in pure Zn and Zn–Mg alloy groups were also significantly increased, while the performance associated with the Zn–Mg alloy was found to be superior to that associated with the pure Zn extract. This could be explained by the Zn–Mg alloy extract stimulating the expression of osteogenesis proteins OCN and RUNX-2, thus promoting osteogenesis. RUNX-2 is a key regulator of osteogenesis differentiation and regulates the expression of many osteogenesis-related genes, including OCN, Col I, and OPN [17]. Moreover, in the early stage of osteogenesis process, some factors also promote RUNX-2 expression to regulate the increase in Col-I [38].

Zhang et al. found that osteoblasts could secrete some osteogenesis factors to further promote the expression of ALP while inhibiting the transcription process of OCN [23,39]. It was also reported that ALP plays an important role in facilitating cell maturation and calcification in early osteogenesis, while OCN promotes the late stage of osteogenesis by binding to minerals, and ALP also promotes OCN expression to some extent in late osteogenesis [38,39,40]. Therefore, the expression of OCN at 14 days was found to be lower than that of other osteogenesis genes. On the other hand, the expression of other genes was higher than that of the control group, while there was no significant difference in OCN expression, which indicates that Zn^2+^ and Mg^2+^ promote osteogenesis at the early stage. Since ALP promotes calcification, and late calcium deposition and mineralization are closely related to OCN, a significant increase in ALP and OCN expression could be observed at 21 days, which is consistent with previous studies [38,39,40]. At 14 and 21 days, no significance difference was observed in the expression of Col I and RUNX-2 compared with the control group, which might explain the slow proliferation of cells in late osteogenesis, where cells were mostly in the mineralization stages. For the immunofluorescence staining results of MC3T3-E1 co-cultured with extract, it can be found that the number of cells in the Zn–Mg alloy group were greater than that of pure Zn and the control groups under the same field of view, and the fluorescence intensity of RUNX2 was also stronger. This suggests that Zn–Mg alloys could promote osteoblast maturation in the early stage. Moreover, the immunofluorescence osteoblast protein expression of Zn–0.5Mg group was higher, which might cause by a synergistic effect of Zn^2+^ and Mg^2+^ to further enhance osteogenesis.

Regarding the angiogenesis property triggered by Zn^2+^ and Mg^2+^, Yang et al. [41] found that the degradation of zinc stents had a promoting effect on arterial remodeling and healing in an in vivo environment. Moreover, Zhao et al. revealed the biphasic effects of zinc ions on human coronary endothelial cells (HCAECs) [38], and there is also a biphasic effect, which is the same as the trend of the effect of Zn^2+^ on endothelial cells studied by Rao et al. [34]. The in vitro studies of J.A.M. Maier et al. showed that a lack of Mg^2+^ inhibits the proliferation and migration of HUVECs [42,43]. Therefore, these results indicate that Mg^2+^ can promote the proliferation of endothelial cells. The concentration of Zn^2+^ in the leaching solution of our experiment is below 100 μM, so the results for the migration, tube formation, and connection experiments are the most optimal for the Zn group (Figure 7A–E). This is because higher concentration of Zn^2+^ promote VEC proliferation, while the results of the Zn–0.5Mg and Zn–1Mg groups are better than those of the Zn–0.2Mg group, which may be related to the effects of Zn^2+^ and Mg^2+^ on endothelial cells.

Studies have shown that HIF-1α levels increase during the early stage and then begin to decline gradually; meanwhile, the peak levels of VEGF in vivo are synchronized with the highest expression of HIF-1α, and the highest levels of VEGF are observed at 3 days [44]. This is mainly because HIF-1α can induce VEGF expression [26], which is also consistent with our RT-qPCR findings (Figure 8A,B). After VEC co-culturing with extract, the expression of HIF-1α and VEGF presented a synchronized tendency between 3 and 7 days, with a higher expression at 3 days than at 7 days. A higher expression was observed in the pure Zn compared with other groups, which is consistent with the results of the migration and tube formation assay, while in the Zn–Mg groups at 3 days, a higher expression of VEGF genes was observed for Zn–0.2Mg compared with Zn–0.5Mg and Zn–1Mg. However, the VEGF expression for Zn–0.2Mg decreased at 7 days, which could be due to the regulatory role of Mg^2+^ in the late stage of vascularization. Therefore, higher Mg^2+^ concentrations can also cause an increase in vascular generation. The promoting effect on angiogenesis and reduced vessel leakage by Mg^2+^ has also been reported in Zheng et al. [45].

Yu et al. [46] developed a dual Zn/Mg ion co-implanted titanium dental implant material via plasma immersion ion implantation. Rapid osseointegration and sustained biomechanical stability are enhanced by the multiple functions co-produced by Zn^2+^ and Mg^2+^. The synergistic effect of Zn/Mg ions is consistent with our research. However, they did not discuss the effects of different contents of Zn^2+^ and Mg^2+^ on the expression of genes related to osteogenesis and angiogenesis. Moreover, by alloying Mg into the Zn matrix in our study, the sustainable release behavior of both ions could be realized in an easy and convenient way, demonstrating biodegradable advantages over traditional bioinert titanium alloys. A similar fabrication method was reported by Liu et al. [47], in which a master alloy was used to incorporate essential element Mg, Na, and Zn ions into the Mg matrix. The co-release of ions in the biodegradable MgSnZnNa alloy resulted in the advanced upregulation of osterix and osteocalcin expression. Furthermore, due to the higher standard electrode potential of Zn (−0.76 V) than Mg (−2.37 V), the current Zn–Mg alloy could present a more suitable degradation rate than Mg-based alloys. In this research, the effects of the biological activities of different Mg contents in Zn-based alloys, as well as the synergistic effects on the expression of related genes in the early and late stages of osteogenic and angiogenic cells, were investigated. Our results could help in providing guidance regarding Zn–Mg composition in designs for orthopedic application from the perspective of biological functions. The flowchart of pure Zn and Zn–Mg alloy extract promoting osteogenesis of osteoblasts and angiogenesis of endothelial cells in vitro during the bone repair process was shown in the graphical abstract.

However, because the complex in vivo environment can never be completely simulated in in vitro experiments, further studies should be performed researching in vivo implantation. Moreover, in silico studies should be considered, since they can offer several advantages, such as faster results and a lack of requirement for sophisticated tools. Further studies of an in silico model based on finite element simulation can be applied in the design and application of orthopedic implants, such total hip implants [48]. At present, there are also studies being conducted on the antibacterial properties that involve inserting bioactive compositions and ions into orthopedic metal substrates, which could offer new insights in designing and fabricating multifunctional biomaterials [49,50].

## 5. Conclusions

In summary, the biodegradable Zn–Mg alloys examined in this study exhibited good cytocompatibility in vitro with osteoblasts and endothelial cells. The release of Zn^2+^ and Mg^2+^ in Zn–Mg alloys was regulated by the addition of different amounts of Mg. Tailoring Mg content not only allowed us to reduce the toxic effect of higher Zn^2+^ concentrations on osteoblasts, but also resulted in the promotion of the proliferation and angiogenesis properties of endothelial cells. Meanwhile, the expression of osteogenesis-related genes and proteins (ALP, COLI, OCN, and Runx-2) was upregulated, and the expression of angiogenesis-related genes (HIF-1α, VWF, VEGF and CD31) was enhanced in the early stage, thereby promoting early bone regeneration. With regard to the two aspects of osteogenesis and angiogenesis, the biodegradable Zn–0.5Mg alloy indicated an ideal bone-promotion performance, with satisfactory biocompatibility and pro-angiogenesis properties. The present work has indicated the potential applications of biodegradable Zn–Mg alloys as candidates of biomedical degradable implants. Moreover, the potential mechanism underlying the beneficial effect of ions in regulating osteogenesis and angiogenesis effects was proposed, which may serve as guidance for the design of biofunctional materials for application in skeletal regeneration. Moreover, future studies of long-term in vivo implantation are still needed, and degradation and the in vivo mechanisms of bone-healing promotion are important subjects that should be studied in future research.

## Data Availability

Not applicable.
